# Bone marrow transplantation for MHC class I deficiency corrects T-cell immunity but dissociates natural killer cell repertoire formation from function

**DOI:** 10.1016/j.jaci.2016.06.029

**Published:** 2016-12

**Authors:** Yifang Gao, Peter D. Arkwright, Rachel Carter, Angelica Cazaly, Rebecca J. Harrison, Alexandra Mant, Andrew J. Cant, Stephan Gadola, Tim J. Elliott, Salim I. Khakoo, Anthony P. Williams

**Affiliations:** a Academic Unit of Cancer Sciences, Faculty of Medicine, University of Southampton National Institute for Health Research Experimental Cancer Medicine Centre, Southampton, United Kingdom; b Department of Immunology, University of Manchester, Manchester Royal Infirmary, Manchester, United Kingdom; c Children's Bone Marrow Transplant Unit, Newcastle General Hospital, Newcastle upon Tyne, United Kingdom; d Academic Unit of Clinical and Experimental Sciences, Faculty of Medicine, University of Southampton, Southampton, United Kingdom

To the Editor:

The examination of primary immunodeficiency represents an opportunity to gain unique insights into models of human immunity.^1^ MHC class I deficiency is a primary immunodeficiency in which both innate and adaptive immune systems are compromised because of the effects of absent MHC class I on CD8^+^ T-cell and natural killer (NK)-cell development and function.^2^ The most frequent cause of MHC class I deficiency is loss of the TAP1 or TAP2 proteins. These proteins import peptides from the cytoplasm to MHC class I molecules within the endoplasmic reticulum, supporting their peptide-loading and cell surface expression. CD8^+^ T cells require MHC class I expression for their thymic selection and development. Mature CD8^+^ T cells target infected tissues by recognizing foreign peptides presented by MHC class I molecules on the surface of infected cells. NK cells also require MHC class I engagement for full functional development during their ontogeny.^3^ However, once fully functional, NK-cell effector functions are inhibited by MHC class I.^4^

We describe the successful outcome and normalization of T-cell immunity after the first allogeneic stem cell transplant for MHC class I deficiency. The child, born in Pakistan to first cousin parents, presented at the age of 6 years with a 2-year history of recurrent *Streptococcus pneumoniae* and *Haemophilus influenzae* chest infections, with radiological evidence of bronchiectasis. From the age of 10 years, she developed chronic, slowly enlarging skin ulcers over the extensor surfaces of both elbows (Fig 1, *A*). Human herpes viruses and EBV were identified from skin biopsies by PCR but blood remained negative for these and other viral types including cytomegalovirus (data not shown). The ulcers failed to respond to antibiotics and antiviral agents. A male second cousin, who had a similar clinical phenotype with bronchiectasis and chronic skin ulcers, died at the age of 10 years of cor pulmonale secondary to severe chronic lung disease.

Initial immune investigations revealed a distinctive phenotype of a high CD4/CD8 ratio (10:1) with low numbers of CD8^+^ T cells (0.29 × 10^9^/L) and normal immunoglobulins (see Table E1 in this article's Online Repository at www.jacionline.org). Because of the child's poor clinical response to conventional treatment, continued deterioration in respiratory function, skin ulcers, abnormal immune function, and family history, she underwent a stem cell transplantation for an undefined immunodeficiency. She was homozygous across the HLA (A2402/−, B3502/−, Cw0401/−, DRB10301/−, DQB10201/−, DPB10501/) and transplanted with a partially matched unrelated female donor (A2407/−, B3503/−, Cw0401/−, DRB10301/−, DQB10201/−, DPB10501/−) at the age of 13 years. This followed low-intensity conditioning with campath 1H 0.2 mg/kg dose (×4), fludarabine 30 mg/m^2^ dose (×5), and melphalan 140 mg/m^2^ dose (×1). Her clinical course posttransplantation was uncomplicated. At 6 months posttransplant, the patient was 100% donor chimeric in both the myeloid and lymphoid lineages. Her clinical condition had improved and the large ulcers had healed (Fig 1, *B*).

In view of her clinical presentation, immunological abnormalities, and homozygosity at the MHC, she was extensively investigated for MHC class I deficiency.

Total surface MHC class I expression (A2402, B3502, Cw0401) on the patient's pretransplant PBMCs showed a reduction in surface MHC class I expression (Fig 1, *C*). This expression was notably higher than that in previous reports from patients with MHC class I deficiencies.^5^ Flow cytometry using locus-specific antibodies for the TAP-dependent HLA-C and HLA-E alleles demonstrated an absence of both alleles (Fig 1, *C*). These 2 alleles are key ligands for NK-cell ontogeny and function. The patient's Cw0401 allele is cognate for the KIR2DL1 receptor while HLA-E is recognized by NKG2A; these are the only cognate MHC-KIR interactions present in this individual. Evaluation of patient's PBMCs using intracellular flow cytometry for TAP1 and TAP2 proteins demonstrated absence of both proteins pretransplant (Fig 1, *D*). Molecular studies demonstrated truncation of TAP1 cDNA after exon 3 and complete absence of TAP2 cDNA. Genomic DNA evaluation identified a homozygous deletion from exon 3 of TAP1 through to exon 11 of TAP2, confirming the first description of a combined TAP1 and TAP2 deficiency (see Fig E1 in this article's Online Repository at www.jacionline.org).

Posttransplant the MHC class I expression returned to normal (Fig 1, *B*) with restoration of TAP1 and TAP2 expression by flow cytometry (Fig 1, *D*). At 6 months posttransplant, the CD8^+^ T-cell population increased to 45% of T cells and the CD4:8 ratio corrected to 1:1 (Table E1). We evaluated the TCR Vβ profile posttransplant to establish the reconstitution of a polyclonal repertoire of T cells. The TCR profile of the posttransplant CD8^+^ T cells at 1 year showed broad representation of all T-cell receptor Vβ family members (Fig 2, *A*). Additional naive T-cell phenotyping showed that 53% of CD4^+^ and 11% of CD8^+^ T cells were naive. T-cell proliferation studies returned to normal, and all parameters were stable over the first 36 months posttransplant (Table E1).

The theoretical concern post–stem cell transplantation for MHC class I deficiency is that donor NK cells will be successfully reconstituted following cognate engagement with MHC class I–competent hematopoietic tissue and this may lead to deleterious self-reactivity with MHC class I–deficient tissue. Post–stem cell transplantation, the NK-cell distribution returned to normal representing 22% of lymphocytes, with 7% of them being CD56^bright^ compared with 46% pretransplant (Table E1). NK cells also displayed a different phenotype posttransplant whereby they lost the unusual phenotype of being multi-KIR positive, dropping from 43% pretransplant to less than 2% of NK cells (Table E1). In addition, the posttransplant NK cells displayed a reconstitution of the expected single positive cognate KIR2DL1:HLA-Cw4 population (Table E1). Despite these changes in NK-cell phenotype, the functional deficiency of the NK cells remained impaired posttransplant, showing no difference to pretransplant NK cells in cytokine and degranulation assays against MHC class I–deficient target cells (Fig 2, *B*).

In MHC class I deficiency, the NK-cell population has not encountered cognate MHC class I ligands and fail to proceed to full functional development.^6^, ^7^, ^8^ Similarly, CD8 T cells have failed to be selected by MHC class I complexes within the thymus, leading to poor development. In the posttransplant setting, hematopoietic reconstitution has supported CD8 T-cell development, leading to naive and TCR diverse populations. For NK cells, there is evidence of engagement with the restored MHC class I competence of the hematopoietic lineage, leading to phenotypic change but this does lead to full functional restoration. This NK-cell hypofunction may protect against self-reactivity to MHC class I–deficient somatic tissue. In summary, this patient has improved dramatically following bone marrow transplant for MHC class I deficiency and now exhibits a distinctive immune status with impaired NK function but restored CD8 immunity that will require long-term follow-up.

## Figures and Tables

**Fig E1:**
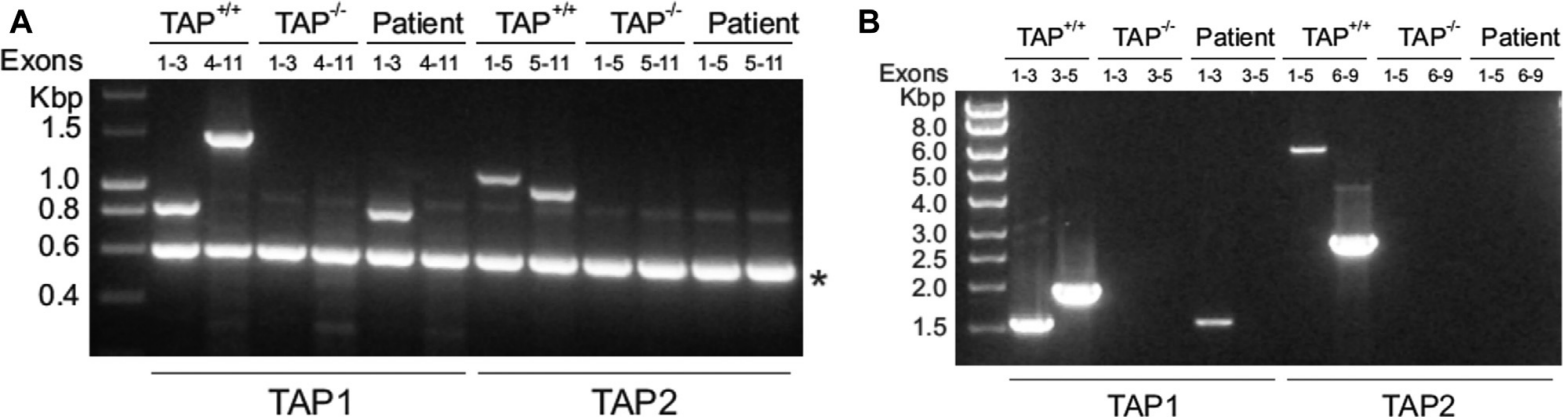
**A,** RT-PCR of the different exons of TAP1 and TAP2 showing absence of TAP1 genes beyond exon 3 and complete absence of TAP2. RNA was extracted from BLCL of the patient, TAP positive (T0) and TAP-negative (T2) control lines. Tapasin (*) was amplified as a loading control. **B,** PCR from genomic DNA showing the presence of TAP1 up to exon 3 and the absence of TAP2 in the same cell lines as in Fig E1, *A*.

**Table E1:** Lymphocyte phenotype and *in vitro* proliferative responses at pretransplantation and posttransplantation time points

Subset	Pretransplant	Posttransplant	Reference range
Lymphocytes (×10^9^/L)	4.2	3.7	1-5
T-cell enumeration			
CD4 (×10^9^/L)	3.0	1.2	0.4-2.1
CD8 (×10^9^/L)	0.29	0.9	0.2-1.2
CD4/CD8 ratio	10	1.3	1-3.6
Naive CD4 (% of CD4+ T cells)	72	53	25-55
Naive CD8 (% of CD8+ T cells)	88	11	10-50
TCR Vβ expression	82	86	>70%
T-cell function			
Unstimulated (cpm)	261∗	288†	1,435∗/376†
Anti-CD3 (cpm)	5,638∗	136.617†	158,306∗/201,034†
Anti-CD3 + IL-2 (cpm)	477,291∗	261,708†	464,012∗/280,034†
PMA (cpm)	7,003∗	125,751†	299,981∗/114,851†
PMA + ionophore (cpm)	380,870∗	177,818†	291,281∗/182,116†
PHA (cpm)	146,845∗	270,906†	259,052∗/271,222†
ConA (cpm)	316,114∗	227,533†	256,313∗/178,131†
NK-cell enumeration (% NK cells)			
NK cells (×10^9^/L)	0.13	0.8	0.07-1.2
CD56^bright^ NK cells (% NK cells)	46	7	1-10
Triple KIR/NKG2A^+^ NK cells	43	<2	<2
Cognate MHC-KIR^+^ NK cells	<2	37	ND

*Con A*, Concanavalin A; *cpm*, counts per minute; *ND*, not determined; *PHA*, phorbol 12-myristate 13-acetate.

∗ Control value for pre.

† Control values for postnaive T cells were identified as CD45RA CCR7+ve. Triple KIR/NKG2A NK cells are represented by those that coexpress KIR 2DL1, 2DL2/3, and KIR 3DL1/NKG2A. Cognate MHC-KIR+ NK cells are represented by those coexpress KIR 2DL1 (cognate for cw0401).

**Fig 1:**
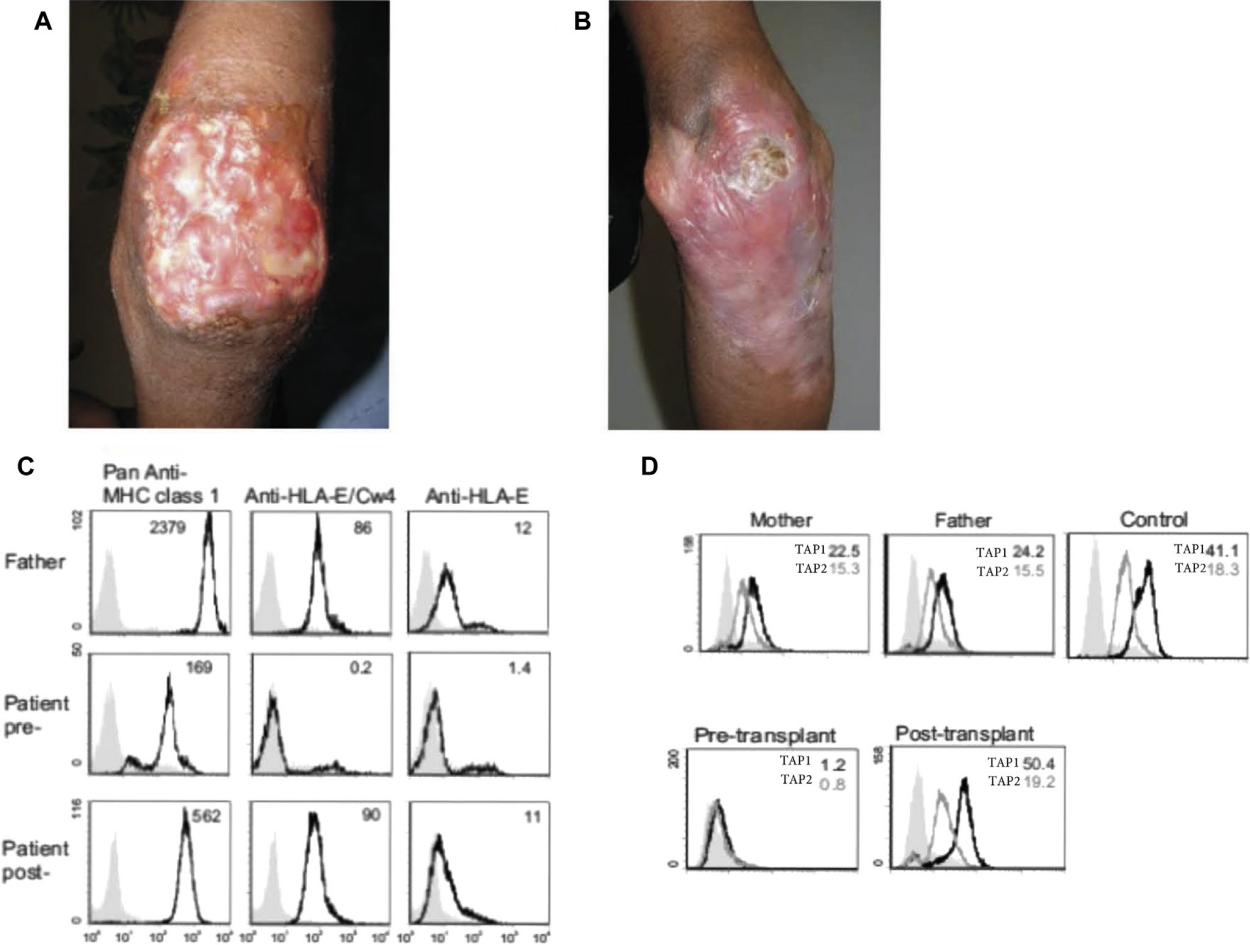
Clinical presentation of the patient showing pretransplant necrotic ulcer of the right elbow **(A)** and posttransplant healed ulcer of the right elbow at 12 months **(B)**. **C,** Flow cytometry of MHC class I expression on PBMCs taken pretransplantation and posttransplantation, compared with that of the father (HLA-A2/24; HLA-B35/61; HLA-Cw4/Cw15). Isotype control (*filled histograms*) and specific anti-HLA antibodies (*black unfilled histograms*) are shown together with the MFI of MHC class I expression. **D,** TAP expression by intracellular flow cytometry of patient BLCL pretransplantation and posttransplantation compared with that of the father, mother, and healthy control. Cells were stained for TAP1 (TAP1.28, *unfilled histograms black line*), TAP2 (TAP2.17, *unfilled histograms gray line*), or relevant isotype control (*filled histograms*). Histograms were gated on live lymphocytes. The MFI of the TAP1 and TAP2 expression is shown. *BLCL*, EBV transformed lymphoblastoid B cell line; *MFI*, mean fluorescence intensity.

**Fig 2:**
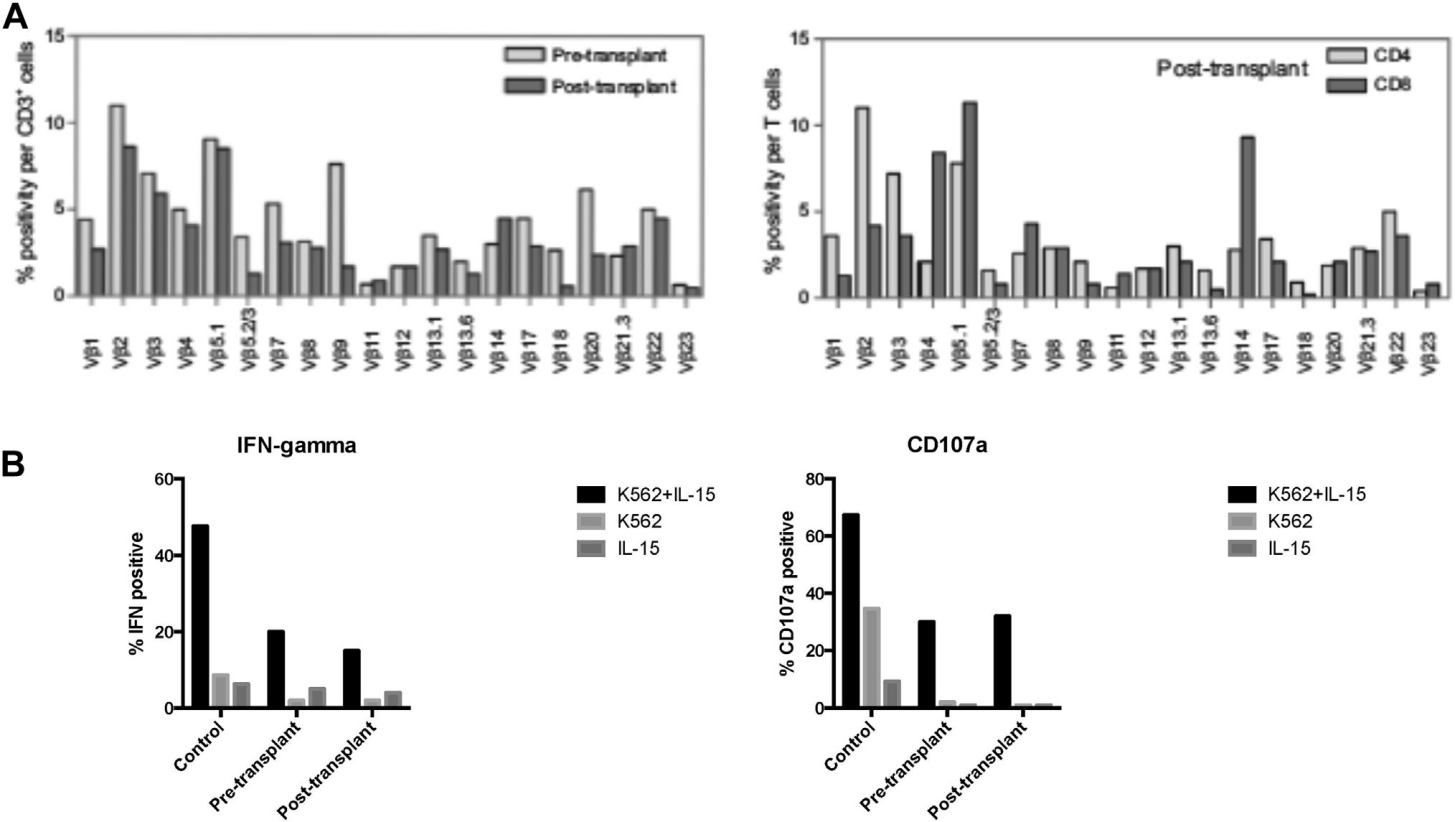
**A,** Flow cytometric analysis of T-cell receptor Vβ family expression on pretransplant and 12-month posttransplant T cells (*left panel*) and on the CD4 or CD8 T-cell subsets posttransplant (*right panel*). The frequencies of the individual Vβ family within each subpopulation are shown as a percentage of that particular subset. **B,** Comparison of NK-cell function pretransplant and posttransplant (18 months) with 3 healthy controls. Shown are the percentages of CD3^−^veCD56^+^veNK cells expressing either IFN-γ by intracytoplasmic cytokine staining or degranulating (CD107a expression) in response to IL-15, K562 or IL-15 and K562, as determined by flow cytometry.
